# Is an Apicomplexan Parasite Responsible for the Collapse of the Iceland Scallop (*Chlamys islandica*) Stock?

**DOI:** 10.1371/journal.pone.0144685

**Published:** 2015-12-18

**Authors:** Árni Kristmundsson, Ásthildur Erlingsdóttir, Mark A. Freeman

**Affiliations:** 1 Institute for Experimental Pathology at Keldur, University of Iceland, Fish disease Laboratory, Reykjavík, Iceland; 2 Institute for Oceanic and Earth Sciences, University of Malaya, Kuala Lumpur, Malaysia; 3 Ross University School of Veterinary Medicine, Basseterre, St. Kitts, West Indies; Institute of Oceanology, Chinese Academy of Sciences, CHINA

## Abstract

Due to the total and unexpected collapse of the Iceland scallop, *Chlamys islandica*, stocks around Iceland during the 2000s, a commercial fishing ban has been imposed on this valuable resource since 2003. Following the initial identification of an apicomplexan parasite in the scallops, a long-term surveillance program was established to evaluate the effect of the parasite on the population. The infections were highly prevalent in all shell sizes throughout the study. However, the parasite only impacts mature scallops where they cause severe macroscopic changes, characterized by an extensively diminished and abnormally coloured adductor muscle. A highly significant relationship was observed between infection intensity and gonad and adductor muscle indices. The first four years of the study, were characterized by high infection intensity and very poor condition of the adductor muscle and gonads, whilst during subsequent years, infections gradually decreased and the condition of the scallops improved. Histopathological changes were restricted to the presence of apicomplexan zoites which were widely distributed, causing varying degrees of pathology in all organs. In heavy infections, muscular and connective tissues were totally necrotized, destroying significant parts of numerous organs, especially the adductor muscle, digestive gland and gonads. The progression of the disease was in good synchrony with the mortality rates and the subsequent decline observed in the scallop stock and recruitment indices. Our findings strongly suggest that the apicomplexan parasite played a major role in the collapse of the Iceland scallop stock in Breidafjordur. In addition to causing mortality, the infections significantly impact gonad development which contributes further to the collapse of the stock in the form of lower larval recruitment. Furthermore, compelling evidence exists that this apicomplexan pathogen is causing serious disease outbreaks in other scallop populations. Similar abnormal adductor muscles and the parasite itself have been identified or observed in association with other mass mortality events in several different scallop species and commercial stocks in the northern hemisphere.

## Introduction

Iceland scallop, *Chlamys islandica* (Muller, 1776) (Mollusca: Pectinidae) is a cold water bivalve species inhabiting the boreal-subarctic transition zone. In the NE Atlantic it is found around Iceland, in the Barents Sea and from the northern part of Norway, south to Bergen and the Stavanger Fjord. In the NW Atlantic, it is found in West Greenland, from Thule in the north to Cape Farewell in the south and along the eastern coast of Canada, from Cumberland Peninsula, Hudson Bay and south to Massachusetts [1].

Commercial fisheries of Iceland scallop have mainly been in Icelandic waters, but also on a smaller scale off West Greenland, in the Barents Sea and off the Atlantic coast of North America [1]. The fisheries in Iceland date back to 1969 with the main fishing grounds in Breidafjordur West Iceland, constituting 60–100% of the total catch, with the scallop industry forming a vital part of the economy in that region [2]. Other fishing grounds of less importance are the Westfjords in NW Iceland, Húnaflói in northern Iceland and Hvalfjordur in the southwest. From 1980 to 2000, the annual catch ranged from 8,500–17,000 tn, reaching a peak in the years 1983–1986 followed by a stable 8,000–9,000 tn catch from 1990–2000 [3].

The Iceland scallop is a relatively slow growing and long-lived bivalve species, its maximum observed age being 23 years [4]. It is dioecious (separate sexes) [5] similar to the Atlantic sea scallop, *Placopecten magellanicus* [6], while many species in the scallop family are hermaphroditic, such as the king scallop, *Pecten maximus* [7] and queen scallop, *Aequipecten opercularis*, [8]. The most commercially valuable part of the scallop is its large adductor muscle although gonads or even whole scallops are also utilized to some extent. In Iceland, the scallop fisheries are carried out by dredging and mostly just the adductor muscle is utilized and marketed as a high value sea food product [9].

In autumn 2000, the first indications of abnormalities in the scallop stock in Breidafjordur appeared, when diminished and abnormally coloured adductor muscles were noticed during processing. Subsequently, evidence of natural mortalities (not fishing-associated) in the stock emerged in 2001 when high numbers of cluckers (empty shells still attached by the hinge) were observed, which were almost exclusively restricted to mature scallops (shells > 5 cm). These high natural mortalities were confirmed not to be associated with fishing pressure, as they were, in many cases, observed at scallop grounds where little or no fisheries had been undertaken [3]. Surveys of scallop stocks in Breidafjördur, the main scallop grounds in Iceland, over the years 2000–2006, performed by the Marine Research Institute (MRI) in Iceland, showed an 84% decrease compared to its average size in the years 1993–2000 [10]. In the years 2007 and 2008 the stock index reached a historical minimum, being merely 13% of its average size in the 1990s [11]. At other scallop grounds around Iceland a similar pattern was also emerging: in the Westfjords in the northwest, Húnaflói in the north and Hvalfjordur in the southwest, where the scallop stocks collapsed despite little or no fishing in these areas [12]. Due to these findings, no scallop fishing has been allowed in Icelandic waters since 2003.

In November 2002, when the downtrend in stock abundance became evident, live scallops were sent to the Fish Disease Laboratory at the Institute for Experimental Pathology at Keldur for examination. This initial examination of affected scallops revealed the presence of two apicomplexan parasites, one of which was found infecting muscular tissues and the other, *Margolisiella islandica*, the heart auricles [13,14]. Following the initial identification, a monitoring program was established where samples from affected scallop beds were examined for these infections at regular intervals. These examinations have shown that infections of *M*. *islandica* in the heart auricles are equally prevalent and intense in all shell sizes and that they do not negatively affect the scallops (unpublished data). Here we present the results of this survey, which now spans 12 years, with regards to the apicomplexan species infecting the adductor muscle [13] its infection prevalence and intensity and impact on the condition of the stock of the Iceland scallop in Iceland.

## Materials and Methods

### Sampling

The field studies did not involve endangered or protected species. All the sampling sites are a property of the Icelandic state and no specific permissions were required for these locations/activities. The Marine Research Institute (MRI– www.hafro.is), responsible for all sampling, was established in 1965 and is working under the direct authority of the Ministry of Industries and Innovations in Iceland and conducts various marine research and provides the Ministry with scientific advice based on its research on marine resources and the environment, according to specific legislations (1965 nr. 64—see: http://www.althingi.is/lagas/140a/1965064.html).

**Fig 1:**
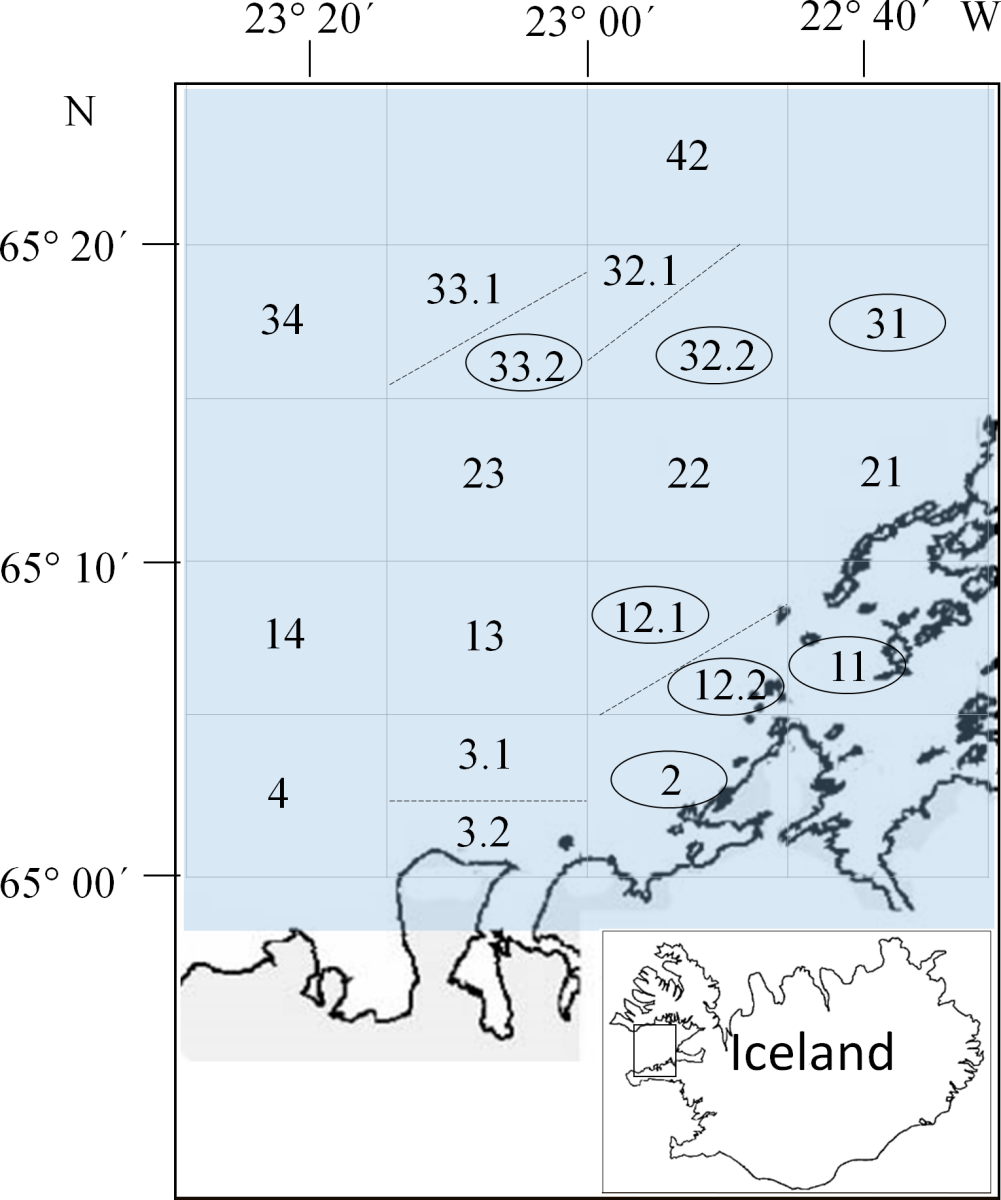
The research site; Breidafjordur, Iceland. The numbered squares represent all the main fishing grounds in the area. The encircled numbers are the sites from which scallops were sampled. Details of samples collected at each site are shown in S1 Table.

During routine expeditions by the MRI in Iceland in the years 2003–2014, a total of 1493 scallops (size range 1.5–9.1 cm) were sampled from seven different sites in Breidafjordur in West Iceland (Fig 1). The scallops were sampled by dredging from natural beds of populations suffering extensive natural mortality. In the years 2003–2006, a mixture of all shell sizes were sampled (N = 637) but from 2007 to 2014 (total N = 854), almost exclusively sexually mature shells (5 cm or larger) (N = 847) were taken. The sampling times and sites and the number of scallops collected are shown in S1 Table.

### Scallop examination

All scallops were brought live to the laboratory and held in seawater until examined, which was within 48 h of collection. When dissected, the internal organs were removed from the shells, the shell height measured (cm) and the wet weight (g) determined for gonads and adductor muscles. Macroscopic changes of the adductor muscles were graded on the scale from 0–3 where: Grade 0 = normal muscle; Grade 1 = muscle light coloured but less compact and with increasing fluid content; Grade 2 = greyish/light brown coloured and loosely bound with high fluid content; Grade 3 = dark grey or brown, very loosely bound with high fluid content and visible holes or hollow areas in the muscle when cut in half.

For each scallop, a tissue imprint from the adductor muscle was made by cutting it in half and pressing their inner side to a microscopic slide. Subsequently the slides were air dried, fixed in methanol for 3 min, stained with May–Grünwald–Giemsa and mounted in resin based medium. The intensities of infections were determined by calculating the mean number of apicomplexan zoites present in 10 microscopic fields from the tissue imprints at 250x magnification. Six of the fields were randomly selected, however due to uneven distribution of the parasite in the adductor muscle; four of the fields were selected where apicomplexan zoites had accumulated. The levels of infections were graded on a scale of 1–5 where: Grade 1 = ≤ 20 zoites per microscopic field; Grade 2 = 21–50 zoites per microscopic field; Grade 3 = 51–100 zoites per microscopic field; Grade 4: 101–200 zoites per microscopic field; Grade 5: > 200 per microscopic field.

### Handling of data

Fulton´s condition index was determined for adductor muscles (MI) and gonads (GI) using the following formulas:

$$MI=100\frac{MW}{SH^{{}^{3}}}$$ (i)

and

$$GI=1000\frac{GW}{SH^{{}^{3}}}$$ (ii)

SH is the shell height (cm), MW is the wet weight (g) of the adductor muscle and GW is the wet weight (g) of the gonads. To make the figures comparable to available data on gonad weight of 7–8 cm scallops in 1988–1990 [15], when the population was considered to be in a normal condition, the weights of the gonads of all fully mature scallops sampled in autumn and spring (≥ 5 cm; n = 1170) sampled were extrapolated to shell size of 7.5 cm with the formula:

$$GW\;=\;\frac{7.5^{3}GI}{1000}$$

Parasitological examination was performed on all 1493 scallops sampled. However, to be able to achieve our goals, three different approaches were applied: 1) Relationship of scallop size/maturity to infection. All scallops sampled in 2003–2006 (N = 637) were split into three size groups and compared, i.e. (i) immature scallops–height less than 4.0 cm (N = 166); (ii) pre-mature and mature scallops–height between 4.0 and 4.9 cm (N = 100); (iii) all mature scallops–shell height 5 cm or more (N = 371); 2) Difference in infections between seasons. All mature scallops (≥ 5 cm, N = 227) from two selected sites (12.1 and 11 –see S1 Table), sampled in spring (n = 114) and autumns (n = 113) in the years 2005–2006, were analysed; 3) Progress of infections and macroscopic changes and their relationship with the muscle and gonad indices (MI and GI) of all mature scallops (≥ 5 cm) sampled during 2003–2014 (N = 1218).

### Histopathology

To study the histopathological effect of infection, all major organs from 200 scallops, from normal-looking to ones with variable degree of macroscopic changes in the adductor muscle, were fixed in Davidson’s fixative [16] for 48 h and subsequently dehydrated in 70% ethanol and processed according to routine histological protocols. Giemsa stained sections (4 μm thick) were examined for histopathological changes related to infections. Furthermore, small pieces (≈ 1 mm^3^) of muscles from five heavily infected scallops were fixed in 2.5% buffered glutaraldehyde in 0.1 M sodium cacodylate buffer (pH 7.4) for 24 h at 4°C, washed three times in cacodylate buffer, post-fixed for 1 h in 1% OsO4, rinsed again with buffer, dehydrated in a graded alcohol series. Subsequently, semithin sections (0.5 μm thick) were cut and stained with toluidine blue and mounted in resin based medium.

### Transmission electron microscope (TEM)

Muscles samples from 20 scallops with severe macro- and histopathological changes were processed as described for the semithin sections in the histopathology section above. Ultrathin sections were cut, stained and examined for the presence of viral particles associated with histopathological changes in the muscle, using a FEI, Tecnai G2 Spirit Biotwin TEM.

### Molecular work

Freshly dissected adductor muscles, from both normal-looking scallops and individuals showing typical gross signs of infections, were fixed in 95% ethanol for molecular analysis; five samples from each sampling site whenever sampled (total number of samples = 180). Total DNA was extracted using a GeneMATRIX kit (EURx Poland) following the tissue protocol. Apicomplexan small subunit ribosomal DNA (SSU rDNA) was amplified from the parasite using the primers and PCR conditions as previously described by Kristmundsson et al. [14]. In addition, the primer pairs 18e / SC2-1370r 5' tccttcatatgtctggcactag 3' and SFC-1120f 5'gaacgaaagttrggggmtcg3' / 18gM [17] were used following the same PCR protocol. From the initial sequence reads two more specific primers were designed from related apicomplexan sequence alignments to be used as a diagnostic PCR for this apicomplexan; 18e-Mer 5' ctgccagtagttatacgt 3' and Mer-790r 5'acacscttgaagcaccctac amplify a 772 bp section on the 18S including the variable v1-v4 regions. PCR conditions were as previously described but used an annealing temperature of 64°C with an extension time of 30 s.

PCR bands of the expected sizes were recovered from the PCR products using a GeneMATRIX PCR extraction kit (EURx Poland). All PCR reactions were performed in triplicate (five infected scallops). Sequencing reactions were performed using BigDyeTM Terminator Cycle Sequencing chemistry utilising the same oligonucleotide primers that were used for the original PCRs. DNA sequencing was performed in both forward and reverse directions for all PCR products and nucleotide BLAST searches performed for each sequence to confirm an apicomplexan origin. The contiguous sequence was obtained manually using CLUSTAL_X and BioEdit [18].

### Terminology and statistics

Ecological terms are according to previous definitions [19]. All statistical tests and plots were performed using RStudio (version 0.98.1062). See S1 Statistics) for an overview of all statistical analyses applied in the study.

## Results

### Shell size/maturity

**Fig 2:**
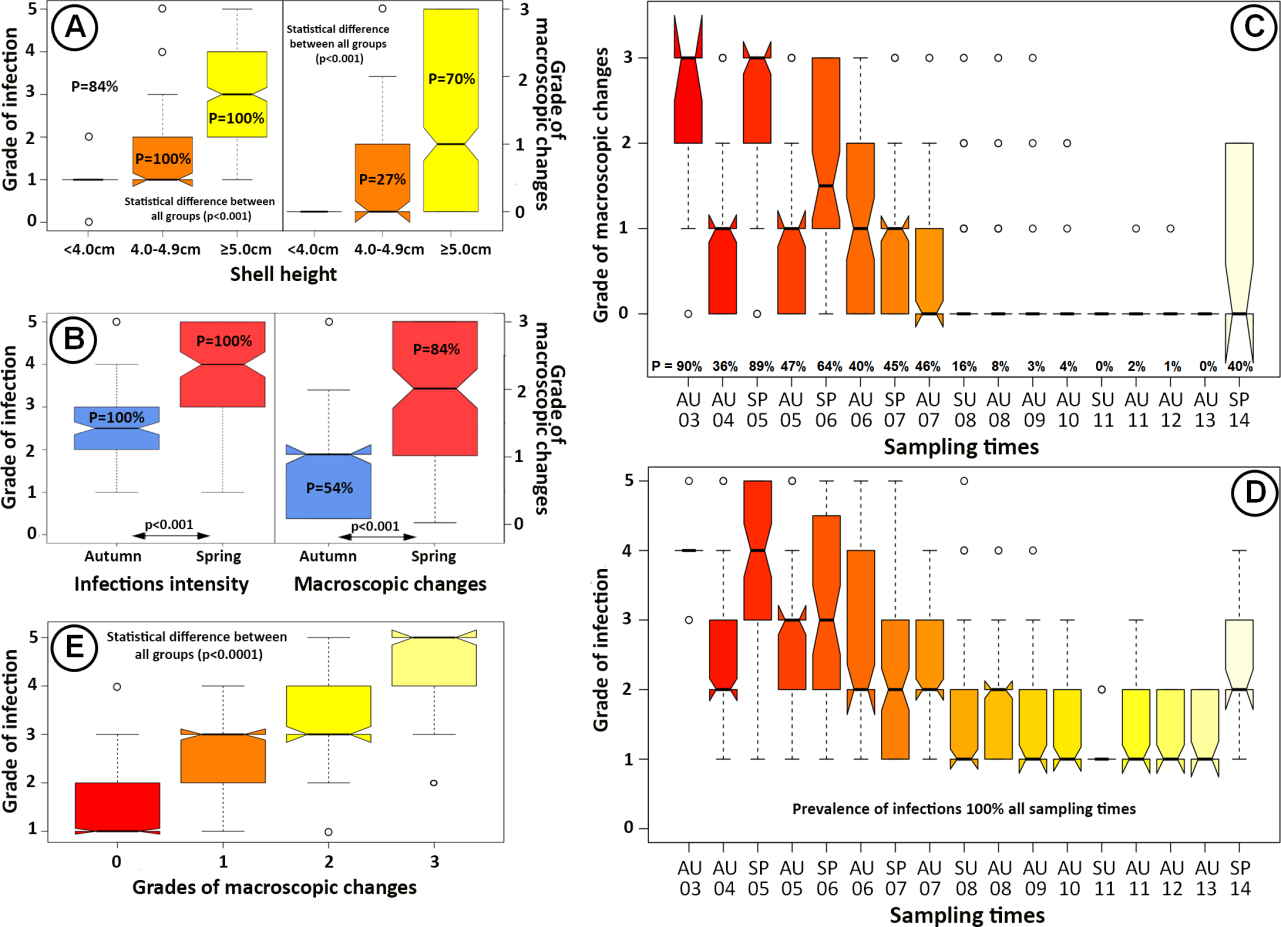
Seasonal and size difference and infection´s progress in mature scallops. (A) Comparison of infections between three size groups of scallops sampled in 2003–2006 (n = 637). The prevalence of infections (P) is high in all size groups. No macroscopic changes are visible in immature scallops (< 4.0 cm; N = 166), while 27% of those reaching maturity (4.0–4.9 cm) (N = 100) have macroscopic signs and 70% of fully mature scallops (≥ 5.0 cm; n = 371). Both the grades of infections and macroscopic signs increase with maturity/size of the scallops and the differences between the median values highly significant between all size groups, both for macroscopic changes and infections intensity. **(B)** Comparison of the seasonal variation in prevalence and median grade of infections and macroscopic changes of 227 scallops (spring = 114; autumn = 113) sampled from two different sites (12.1 and 11) in 2005 and 2006. The prevalence of macroscopic changes is considerably higher in the spring (84%) than in the autumn (54%). The median grades of infections and macroscopic changes are significantly higher in the spring in all cases (p < 0.0001). **(C and D)** The prevalence (P) and severity of macroscopic changes (C) and infections (D) for all mature scallops (≥ 5cm) sampled in 2003–2014 (N = 1218). Both the prevalence (P) and the grades of macroscopic changes and infections are most prominent from 2003–2006 with a subsequent gradual decrease from 2007–2013. In 2014, an increase is observed; however, in most cases the grades of the macroscopic changes are mild. AU = autumn; SP = spring; SU = summer. *The notches on the boxplots provide an approximate 95% test of the null hypothesis that the true medians are equal: if the notches do not overlap, the medians could be described as statistically significant.

The prevalence of infection, determined by examination of stained imprints, was high in all size groups; 100% in size groups 4.0–4.9 cm and > 5 cm and 84% of shells < 4.0 cm. Macroscopic changes in the adductor muscle varied considerably between the size groups as none were observed in < 4.0 cm group, 27% in the 4.0–4.9 cm group and 70% in shells larger than 5.0 cm. Furthermore, the median grade of macroscopic changes was significantly higher (p < 0.0001) in the **>** 5.0 cm group than in the 4.0–4.9 cm group. Similar to macroscopic changes, the grades of infection (examination of stained imprints) was highest in the largest scallops and lowest in the smallest ones with highly significant differences (p < 0.0001) between all groups (Fig 2A).

### Seasonal difference

A seasonal difference was observed in both prevalence and mean grades of macroscopic signs and mean intensity of infection. For both sampling sites and years tested for this purpose (sites 12.1 and 11), the prevalence and macroscopic changes in the adductor muscle were considerably higher in the springtime than in the autumn. Furthermore, both the grades of infections and macroscopic changes in the adductor muscles were significantly higher in spring (p < 0.0001) (Fig 2B).

### Mature scallops; progress of infections and macroscopic changes

All mature scallops were found to be infected throughout the study. From 2003–2007, the occurrence of macroscopic changes in the adductor muscle was high, ranging from 36–90% with a subsequent decrease to 8–16% in 2008. During the following five years, the prevalence of macroscopic changes was low, not exceeding 4% but increased to 40% in a limited numbers of scallops from one site in spring 2014. The median grade of macroscopic changes varied somewhat between sampling times being highest the first four years and more severe in the springtime, with the exception of autumn 2003. Conversely, although 40% of the scallops showed macroscopic changes in spring 2014, they were commonly light and only of grades 1 and 2 (Fig 2C). The grades of infection showed a similar pattern to the prevalence and severity of macroscopic changes, i.e. being high the first 4–5 years of the survey, followed by a gradual decrease over the next six years and a sudden increase in spring 2014 (Fig 2D). A highly significant positive relationship (p < 0.0001 between all groups) was evident between the grades of infections and macroscopic changes (Fig 2E).

### Effect on the condition of mature scallops

**Fig 3:**
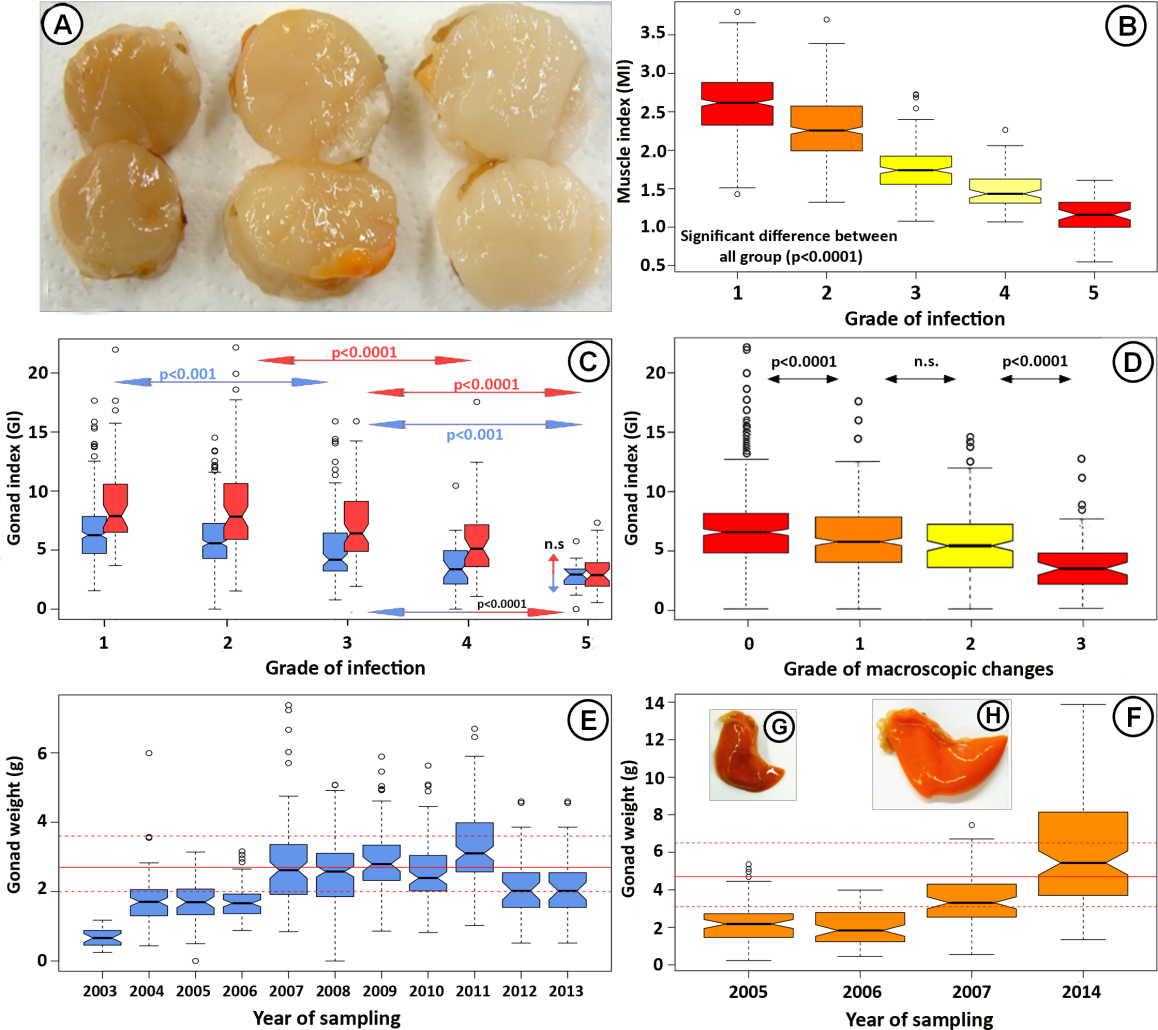
Effect of apicomplexan infections on the scallop condition. (A) Adductor muscles from three mature scallops. The first on the left is from 7.8 cm scallop which has severe macroscopic changes (grade 3; MI = 1.4), it is brownish coloured and greatly diminished and extensively infected while the first on the right is from 7.2 cm healthy looking one with firm and light coloured adductor muscle (MI = 2.5) which had a mild apicomplexan infection. The one in the middle is from a 7.5 cm scallop with grade 1 macroscopic changes (MI = 1.9). (B) The relationship of the MI and the grades of infections from all mature scallops examined (N = 1218). The MI deceases significantly with increasing grades of infection (p < 0.0001 between all grades). (C) The relationship of the GI and the grades of infections from all mature scallops sampled in the spring (red boxes; N = 297) and autumn (blue boxes; N = 794). A reduction in the GI is apparent, especially in the spring (red double arrow line). Note that in spring the GI should be much higher as these shells should be close to full maturity. However, scallops with grade 5 infections is not significantly different between autumn and spring. Furthermore, the GI for grade 5 spring scallops is significantly lower than the one for grade 1–3 autumn scallops (blue/red double arrow line). (D) The relationship of the GI and the grades of macroscopic changes observed in adductor muscles. The GI significantly decreases with severity of the macroscopic changes. (E and F) Comparison of the gonad weigh of 7.5 cm scallops caught in autumn (E) and spring (F) in 1988–1990 [15] and 2003–2014 (extrapolated to 7.5 cm). The whole and broken horizontal red lines represent the normal mean range of gonad weight observed by Thorarinsdottir [15] from a healthy population of scallops in 1988–1990. (E) Scallops sampled in autumn (N = 794). The gonad weight of scallops from 2005 and 2006 is greatly reduced but from 2007 their weigh is similar to those observed in 1988–1990. (F) Scallops sampled in spring (N = 297). The gonad weight of scallops from 2005 and 2006 is greatly reduced. In 2007, their weigh is approaching normal and from that time their weight is similar to those in 1988–1990. (G) Macroscopic changes observed in the gonad caught in the spring and (H) healthy looking gonad caught at the same time. *The notches on the boxplots provide an approximate 95% test of the null hypothesis that the true medians are equal: if the notches do not overlap, the medians could be described as statistically significant.

The most apparent macroscopic changes observed were greyish/brownish and greatly diminished adductor muscles compared to the normally sized, light coloured normal-looking ones (Fig 3A). Infections severely affected the condition of both the adductor muscle and the gonads (using Fulton´s condition index; MI = Muscle index and GI = Gonad index). A significant negative relationship was observed between all different grades of infections and the MI (p < 0.0001 in all cases) (Fig 3B). Macroscopic changes were also commonly observed in gonads with low GI. These were diminished and dark compared to the orange coloured normal ones (Fig 3G and 3H). Similar to the MI, the GI decreased with higher grade of infection, both for scallops sampled in spring and autumn, although a significant difference was not observed between all grades of infections (Fig 3C **).** A significant difference was observed between GI for scallops caught in spring and autumn (p < 0.001), except for the most severely infected scallops (grade 5). Furthermore, the GI of spring caught scallops with grade 5 infection (median GI ≈ 3), was statistically lower (p < 0.001) than the GI of autumn caught scallops with grade 1, 2 and 3 (median GI ≈ 6, 5 and 4, respectively) (Fig 3C). In addition, the GI decreased with higher grades of macroscopic signs, being statistically different (p < 0.0001) for all grades, except grades 1 and 2 (Fig 3D).

The gonad weights (extrapolated to 7.5 cm) of all scallops, were compared with analogous data from 1988–1990 [15], when the scallop stock was considered to be normal. The comparison showed that the gonad wet weight in 2003–2006 was much lower than the normal gonad weight, both in spring and autumn. In spring 2007, the weight was approaching normal and from autumn 2007 and till the end of the study it was within the normal range of gonad weight (Fig 3E and 3F).

### Histopathology

Histopathological changes were restricted to the presence of apicomplexan zoites and no pathology was associated with cysts, i.e. gamonts, meronts and oocysts. The apicomplexan zoites were almost exclusively restricted to muscular and connective tissues and the cysts exclusively in the adductor muscles. The apicomplexan zoites were widely spread, causing varying degrees of damage to all scallops examined, depending on the organ infected and the intensity of infections.

#### Adductor muscle and heart

**Fig 4:**
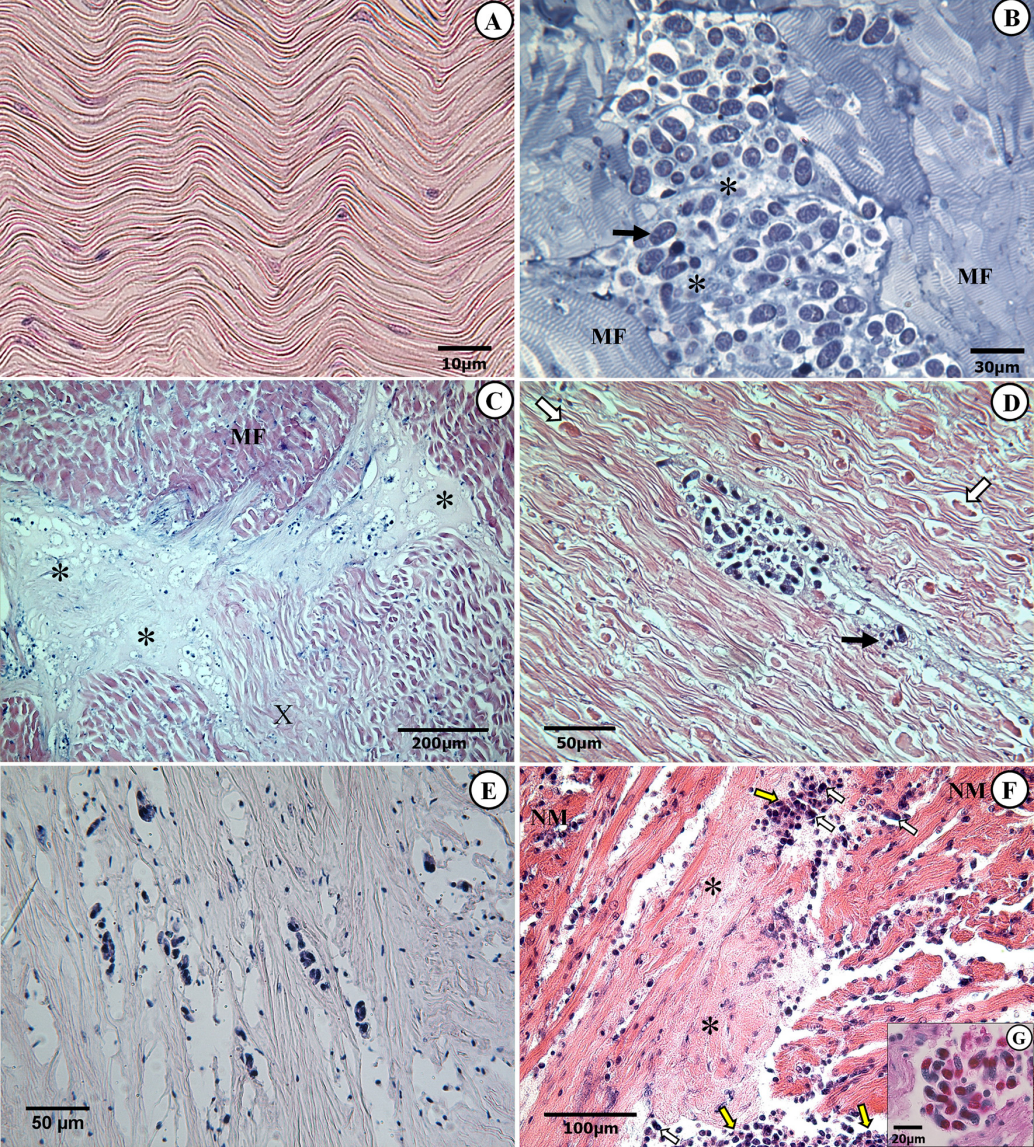
Histopathology of the adductor muscle and heart. Stained histological sections of adductor muscles from heavily infected scallops (B-E) and heart (F and G). (A) A normal striated muscle fibres in a scallop´s adductor muscle. (B) A semithin section showing numerous apicomplexan zoites (black arrow) in the extracellular space associated with necrotic muscle cell debris (*). (C) Severely affected muscle with loss of striation in degenerating muscle fibres (X) and a liquefactive necrosis of large areas (*). (D) A cluster of apicomplexan zoites causing focal necrosis (black arrow) and fragmentation and hyalinization (white arrows) of the surrounding muscle. (E) Necrotized muscle fibres with cytoplasmic vacuolisations, pyknotic nuclei and no well-defined cross-striation. (F) Histological section through a ventricle from an infected scallop showing degeneration of the muscle fibres of the myocardium and infiltration of haemocytes (yellow arrows) mixed with apicomplexan zoites (white arrows); loss of striations in the muscle fibres (*) and muscular necrosis. (G) Higher magnification of a cluster of apicomplexan zoites associated with necrotic cardiac muscle cell debris. Abbreviations: MF = Muscle fibres; NM = Normal muscle.

The parasite was commonly found in large numbers in both parts of the adductor muscle, i.e. the striated phasic adductor and the smaller smooth tonic adductor muscle (catch muscle). In healthy looking scallops the histopathological changes were minor, mostly affecting the adductor muscle in which focal necrosis was present in close vicinity to the apicomplexan zoites. However, in scallops with macroscopic changes in the adductor muscle, severe histopathological changes were observed (Fig 4). Apicomplexan zoites were found in clusters either intracellularly in muscle fibres, extracellular and associated with necrotic muscle cell debris or in the endomysium, a connective tissue surrounding the muscle cells (Fig 4B). Striated and smooth muscles were equally affected. In mild infections of normal looking shells, some light focal necrosis was observed while in the heavily infected scallops, destruction of large parts of the muscle was evident in the form of extensive liquefactive necrosis, a loss of striations, muscular fragmentation and hyalinization (Fig 4C–4E).

Pathological changes were common in the heart ventricle, associated with an accumulation of apicomplexan zoites, especially in the myocardium and also, to some extent, in the epithelial and connective tissue layers of the epicardium. Degeneration of cardiac muscle fibres was common, characterized by loss of striation followed by nuclei degeneration and substantial necrosis. In some cases, a massive infiltration of haemocytes was observed which in some cases formed clusters of hematopoietic centres (Fig 4F and 4G).

#### Gastrointestinal tract

**Fig 5:**
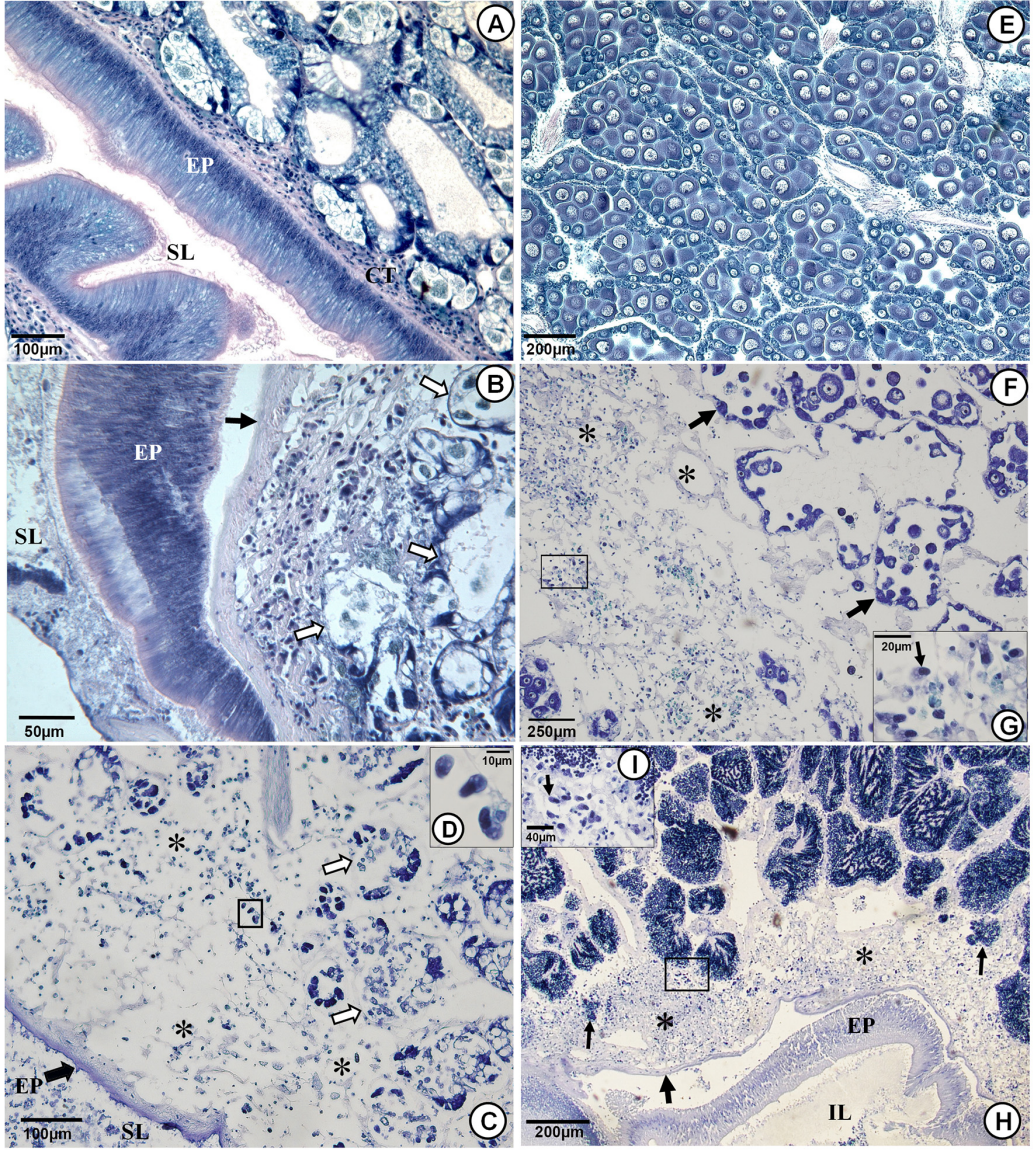
Histopathology of the gastrointestinal tract, digestive gland and gonads. (A) Normal histology of the stomach epithelium and the adjacent digestive gland tubules. Note the firm connective tissue layer (CT) separating the stomach wall and the digestive gland tubules. (B) Histological sections through the stomach and the digestive gland of a heavily infected scallop showing extensive accumulation of apicomplexan zoites in connective tissues surrounding the stomach epithelium and in the digestive gland interstitium causing separation of the basement membrane (black arrow) from the stomach epithelium (EP) and necrosis of the connective tissues and digestive cells (white arrows). (C) A total destruction the digestive gland associated with mixture of cellular debris and numerous apicomplexan zoites (*). The stomach epithelium (EP), connective tissues and digestive cells (white arrows) are heavily necrotized. (D) Higher magnification of the area within the black square of (D) showing apicomplexan zoites. (E and F) Female gonads from scallops caught in early May 2005. A normal one (E), with large acini filled with semi-mature eggs, and a diseased one (F) where the inter-acinal tissues are massively infected with apicomplexan zoites which have caused a total destruction of the acini in large areas (*). The remaining acini have merely premature eggs (arrows) and their development asynchronous with time of year. (G) Higher magnification of the area within the black square of (F) showing apicomplexan zoites (arrow). (H) Section through a heavily infected gonad and intestine of a male scallop. The connective tissue surrounding the intestinal epithelium and inter-acinal tissues are heavily infected with apicomplexan zoites causing the basement membrane to separate from the epithelial lining (broad arrow) and degeneration of the epithelial cells (EP). The apicomplexan causes destruction of the connective tissue which makes up the wall surrounding the acini which subsequently decay (thin arrows). (I) Higher magnification of the area within the black square of (H) showing apicomplexan zoites. Abbreviations. EP = Epithelial layer; IL = Intestinal lumen; SL = Stomach lumen.

Aggregations of parasite zoites were frequently found in loose and muscular connective tissues surrounding the whole gastrointestinal tract, from the buccal cavity, oesophagus, stomach and throughout the intestine. Similarly, the connective tissues surrounding the epithelial lining of the primary and secondary ductus, which connect the stomach to the part of the digestive gland harbouring the digestive cells themselves, were heavily infected as was the interstitial connective tissue surrounding the digestive cells (Fig 5A–5D). The apicomplexan infections were both intracellular in haemocytes and in the extracellular matrix and commonly causing total destruction of these tissues leading to a separation of the basement membrane from the gastrointestinal epithelium. Focal or disseminated necrosis was commonly observed in the interstitial connective tissue surrounding the digestive cells (Fig 5B). In the most severe cases the epithelial layer surrounding the intestine and the surrounding digestive cells was totally destroyed (Fig 5C and 5D). Furthermore, on some occasions, unidentified parasitic forms, possibly growing trophozoites, were observed as intracellular in the gastrointestinal epithelium.

#### Gonads

Extensive aggregations of parasites were routinely observed in both inter-acinal and peri-gonodal connective tissues of the gonads causing disruption in the inter-acinal tissues and a destruction of the wall enclosing the acini, which subsequently lead to the degeneration of primordial germ cells (Fig 5E–5I). In addition to the observed destruction of large parts of the gonads (Fig 5F–5I), the development of the remaining parts of the gonads were asynchronous with season/time, i.e. gonads in scallops harvested in May, when they should be almost fully mature, were only at the initial stages of maturity, normally observed in autumn (Fig 5E and 5F). Similar asynchronous development was observed in scallops sampled in late summer and autumn during the period when infections were most severe (2003–2006). At that time of year, when the scallops are normally in their initial phases of gonad maturation, scallops with a mixture of mature, semi-mature and decaying egg- or sperm acini were observed, indicative of an unsuccessful spawning event.

#### Other organs

Although parasites were normally found in inter-tubular connective and fibromuscular tissues of the kidney, the kidney tubules were usually fairly unaffected. Occasionally some pathology was evident, associated with an infiltration of parasites and haemocytes in the kidney interstitium. These pathological changes were characterized by vacuolization and necrosis of the tubular epithelial cells.

The gills were, in general, lightly affected by the apicomplexan parasite. Although infections were commonly detected, they were characterized by isolated parasites, or groups of a few apicomplexan zoites, which were more or less restricted to muscular tissues in the base of the gill lamellae. The associated pathology was normally mild, characterized by a focal necrosis in the vicinity of the apicomplexan zoites.

A quite variable mixture of tissue types make up the mantle, foot and labial palps, the most common ones being muscle fibres, loose connective tissue, muscular connective tissue and fibromuscular tissues, i.e. a musculature featuring an irregular meshwork of fibrous tissue. Normally the extracellular matrix of the connective tissues in these organs contains high numbers of various types of haemocytes. The outer parts of these organs are enclosed by a lining of epithelial cells. Apicomplexan zoites were routinely found in great numbers in these organs, often causing extensive disruption to all the formerly named tissue types with extensive necrosis associated with the presence of the parasites.

#### Host response

**Fig 6:**
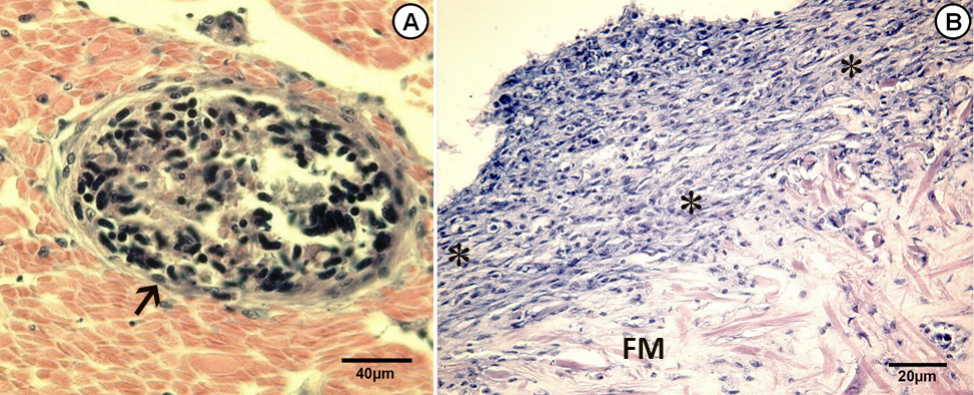
Host responses to infections. Host responses to the apicomplexan infections. (A) An isolation of a cluster of apicomplexan zoites with fibroblast like cells (arrow). (B) Repairing of scallop foot where fibromuscular tissue is substituted by fibroblast like cells (*). NM = Normal muscle; FM = Fibromuscular tissue.

Host responses to infections were generally light. Light infiltration of haemocytes was commonly observed associated with the presence of the apicomplexan zoites. In some cases, especially in the heart myocardium, the foot, the mantle and in the labial palps, the accumulation of haemocytes was extensive. Occasionally, fibroblast like cells were seen surrounding a clusters of apicomplexan zoites (Fig 6A) or developing cysts and on several occasions, in the foot, some signs of tissue repairing were observed, where muscular and fibromuscular tissues were substituted by fibroblast like cells. (Fig 6B)

### TEM

TEM examination of adductor muscles samples, which showed severe macroscopic and histopathological changes, revealed the presence of numerous apicomplexan zoites in association with degenerative muscle fibres and/or muscle cell debris. No viral particles were detected.

### Molecular analysis

A contiguous sequence of 1811 bp of SSU rDNA was successfully obtained for the apicomplexan parasite from infected adductor muscles. BLAST searches showed that the sequence was most similar to those from the apicomplexan genus *Aggregata*, found infecting cephalopod molluscs, with an 81% identity. The diagnostic PCR worked well and was able to successfully detect parasite DNA in adductor muscle samples with no macroscopic changes of infection. The same apicomplexan was detected in all 180 samples tested. No other apicomplexan species were identified in the samples.

## Discussion

### The progression of the scallop stock and apicomplexan infections

The almost complete collapse in the population of the Iceland scallop, *Chlamys islandica*, experienced in Breidafjordur in West Iceland became apparent in the years 1999–2000 [3,20,21] and in 2005–2006 the stock index was only at 16% of its average size from previous years 1993–2000 (MRI, 2007). Concurrently, the recruitment index decreased by approximately 98% [22]. The period from 2000–2006 was characterized by extremely high rates of natural mortality in larger scallops, independent of fishing activities. Associated with these mortalities was a high prevalence of scallops with diminished adductor muscles with an abnormal grey or brown colouration [13,21]. Since that time, no significant changes have been observed in the stock index [10,23].

The observed progression of the apicomplexan infection is in good synchrony with the mortality rates and the subsequent decline observed in the scallop stock and recruitment indices. With respect to the severity of infections and their effect on the condition of the scallops, the first four years of the study (2003–2006), were characterised by high infection intensity and a high prevalence of scallops with abnormally grey or brownish adductor muscles which was loosely bound with a high fluid content and low MI and GI. Conversely, in the latter eight years, a gradual decrease in infection intensity and prevalence and levels of macroscopic changes were observed along with a considerable improvement in both MI and GI. Although the stock index has remained low despite decreasing infections, some signs of recovery are emerging [23,24]. Along with decreasing levels of apicomplexan infections and natural mortality, the proportion of adult scallops (i.e. the affected individuals) have increased significantly, and furthermore, shells exceeding 8.0 cm, which were almost completely depleted, are now found in considerable numbers in the stock. With regards to changes in recruitment, the abundance of juvenile scallops remained poor in the MRI dredge surveys until 2012 when year-classes from 2010 were observed. Furthermore, during the MRI scallop expeditions in 2013 and 2014, juvenile scallops were found in numerous scallop beds, both in the conventional areas and new ones, in considerable amounts; cohorts that will hopefully contribute to the fishing stock in few years time [23,24].

### The effect of infections on the scallop host

Similar to the abnormal natural mortality observed, the negative impact of the apicomplexan infections was restricted to larger mature scallops [3]. Immature scallops were significantly less infected and abnormally looking adductor muscles never observed in scallops of less than 4 cm, whilst a high proportion of the adductor muscles of fully mature scallops (> 5 cm), caught at the same time and site, had macroscopic changes. The Iceland scallop reaches maturity at sizes 4.0–5.0 cm and all scallops below 4 cm are considered immature and all above 5.0 cm fully mature while the sizes in-between include a mix of pre-mature and mature animals [9]. Therefore, the prevalence and grade of macroscopic changes, which correlate with parasite intensity, observed in 4.0–4.9 cm scallops, seems logical. In addition to scallop size, the infection intensity was significantly higher and macroscopic changes more prevalent and intense in scallops caught in the springtime (April–May) than in the autumn (Sept.–Oct.). It therefore seems that size and level of maturity plays a major role with respect to infections and the impact on the host.

The apicomplexan infection severely affects the condition of both the adductor muscle and the gonads. The infection intensity showed a significant positive relationship with the grade of macroscopic changes and negative relationship with MI and GI. Furthermore, the histopathological examination not only showed severe histopathological changes in the adductor muscle and the gonads, but also in most other organs. A comparison of the gonad weight of mature scallops (size 7.5 cm) observed in the current study to those in 1989–1990 [15], when the scallop population was considered to be in a normal condition reflects the decrease in the condition of the scallop stock. The gonad weight during the years 2003–2006 was extremely poor i.e. when the infections were most severe. At this time the gonad weights, in both spring and autumn, were far from normal and in many cases not even half the normal weight of gonads recorded from 1988–1990. In 2007 and 2008 the weights were recovering and approaching normal, and from 2009–2014 their condition had returned to normal. This is fully consistent with the progress of infection intensity, the prevalence of macroscopic changes as well as the natural mortality and stock index during these years. As in other animals, gonad maturation in scallops requires a significant metabolic input. In the case of the Iceland scallop, the gonad development depends largely on energy reserves, such as carbohydrates and proteins from somatic tissues, especially from the adductor muscle, but also other organs such as the digestive gland [25]. The results of the present study show that infections cause varying degrees of pathology in more or less all organs of the Iceland scallops. However, muscle tissue is the main target tissues of the parasite, where it is commonly found in high abundance. Heavy infections cause severe myodegeneration with a total destruction of large parts of the muscle, characterized by severe pathology in both striated and smooth muscle fibres. In addition to causing direct pathology in the gonads, the bad condition of the adductor muscle would further contribute to the poor gonads condition and hamper its normal development, as a result of a lack of stored energy required for normal gonad maturation. Indications of a delayed or failed gonad development and spawning were routinely observed in this study, where scallops harvested in May, when they should be almost fully mature, were merely in the initial developmental stages, and in August–October, some scallops had a mixture of mature, semi-mature and decaying egg or sperm sacs. A good example how severely the gonads were affected by the infections, the GI of spring caught scallops with grade 5 infection (median GI ≈ 3), was statistically lower than the GI of autumn caught scallops with grade 1, 2 and 3 (median GI ≈ 6, 5 and 4, respectively). Under normal conditions this should naturally be the opposite, i.e. around 11 (on average) in the spring compared to 6 in the autumn. The fact that the reduction in the recruitment index exceeded the decline in the stock index support that some of the remaining mature scallops did not contribute to the recruitment due to spawning failure. Examples exist from dredge oysters, *Ostrea chilensis*, where severe apicomplexan infections affected normal gametogenesis by causing a total destruction of the connective tissues surrounding the gonad acini [26], similar to the scallops in the present study.

### Unresolved mass mortality events and abnormal condition of scallop populations

Many examples exist of mass mortality events in wild populations of Iceland scallop, both abroad and in Icelandic waters. Most commonly, these mortalities have been attributed to increasing sea temperature and/or overfishing. However, these conclusions are in most cases highly speculative and the potential role of pathogens and diseases not even considered as no examinations were performed [1,5,9].

In the year 1983, a mass mortality of Iceland scallop occurred in Hvalfjordur in SW Iceland [9]. The reasons for this were unclear but the effect of unusually high sea temperature the previous year was speculated as the cause. In 1985, previously unknown scallop beds were found in several fjords and bays in eastern Iceland [9] and subsequently fisheries were conducted in that area in 1985 and 1986 [21]. However, a research survey in 1998 revealed that these previously commercially valuable scallop beds only consisted of dead shells [27]. In both the above mentioned mortality events, the reasons remain unknown. In either cases, the possible role of infectious agents were not considered as no such research was performed. Similar examples of mass mortalities exist from other northern areas. In 1963, Wiborg [5] reported an apparent extinction of numerous scallop populations in Norwegian waters; scallop beds which only consisted only of empty shells (cluckers). He suggested that changes in environmental conditions, such as a sudden rise in sea temperature, were the cause. In the early 1980s, extensive beds of Iceland scallops had been discovered in the Barents Sea, in the Jan Mayen and Spitzbergen areas and the potential for scallop fisheries was considered to be very promising [1,28]. However, Sundet [28] noted that high percentages of dead scallop shells in the catches would make fishing methods less efficient. Surveys in this area in 1986 [29] indicated further favourable prospects for scallop fisheries. However, as soon as 1987, some of the major scallop beds in the Barents Sea had already collapsed [1], with the cause given as overfishing. A similar situation occurred in the scallop fisheries in Greenland, where massive declines or total losses of scallop beds were experienced, despite limited fisheries of about 10% of the stock size [1]. In addition, considerable decreases in gonad and muscle yield have also been reported from scallop populations around Greenland [1]. Mass mortality of scallop populations have also been reported from the NW Atlantic Ocean, in both Iceland scallop and sea scallop (*Placopecten magellanicus*) stocks in Canadian waters. The causes for these abnormal mortalities could not be determined [30].

In addition to mass mortality events, macroscopic changes similar to those observed in the Icelandic scallop populations have been reported from other populations of Iceland scallop as well as in other scallop species from different geographic areas, including the Barents Sea, NW Atlantic and NE Pacific Ocean [31–33]. In 2006, a disease only affecting mature larger Iceland scallop, was reported from Svyatoy Nos, Russia. The macroscopic changes were dull grey coloured adductor muscles and changes in colour of the gonad. Histopathological investigation were performed which showed severe necrotic changes in the mantle, adductor muscle and gonads [31]. At that time, the associated mortalities had not been determined and to our best of knowledge, the cause for this condition is still unknown. Similar macroscopic changes in adductor muscles were also reported by Kristmundsson et al. [13] from a wild population of queen scallops (*Aequipecten opercularis*) from the Faroe Islands which were associated with heavy infections of the same apicomplexan affecting the Iceland scallop. In the NW Atlantic, darkened and reduced adductor muscles, termed “grey meat”, also restricted to larger Atlantic sea scallops, *Placopecten magellanicus*, have periodically been reported since 1949 [32,34–37]. Recently, large numbers of “grey meat” scallops have been observed in the rotational management areas of Georges Bank after extended fishing closures [38,39]. In 2013, access to certain fishing areas on Georges Bank ended early due to the high number of “grey meat” scallops landed, which have a low meat yield and the discoloured appearance reduces market value [39]. The cause for these mortalities and macroscopic changes is unidentified although effects of senescence and parasitism by shell borers and prokaryotic infections have been suggested as a cause [31,37]. The prokaryotic infections reported by Gulka et al. [37] have been cited as a possible cause by many authors. Gulka et al. [37] reported a sudden and total extinction of a population of sea scallops in Narrangansett Bay, Rhode Island in 1979–1981. Associated macroscopic changes were greyish, flaccid adductor muscles and histology showed extensive myodegeneration. Gulka et al. [37] identified prokaryotic infections in 88% of 34 animals, in gills, plicate membranes and other epithelial surfaces of the body. Associated histopathology was characterized by fragmentation of muscle fibres, hyaline change with loss of cross striations and necrosis with foci of amoebocytic accumulations. When examining Fig 4 in this paper [37], which shows the histopathology in the adductor muscle, many of the structures referred to as amoebocytes within the necrotic area look remarkably like apicomplexan zoites, similar to those observed in the Iceland scallops in the present study. Furthermore, no prokaryotic infections were observed in the adductor muscle where the most extensive pathology was observed. Gulka et al. [37] stated that the heavy gill infection and extensive myodegeneration suggested that gill dysfunction caused metabolic stress causing pathological changes in muscle tissue. However, recently the apicomplexan parasite described infecting the Iceland scallop was identified in sea scallop from both US and Canadian waters, by both morphological and molecular methods [38,39]. In the NE Pacific Ocean and the Bearing Sea, the weathervane scallop, *Patinopecten caurinus*, has been harvested commercially since 1967. Poor quality adductor muscles, termed “weak meat”, characterized by an off-white to greyish colour, with a notable stringy texture, and a spongy consistency causing difficulty marketing them, has resulted in an underutilization of the resource in the eastern Gulf of Alaska [33]. The authors discuss the similarity of this phenomenon to that in the sea scallop, *P*. *magellanicus*, on the eastern coast of North America and the potential causes named in that scallop species, i.e. clionid infestation, prokaryotic infection, and age-related senescence [32,33,37,40,41] but suggest further exploration is required on potential impact of environmental factors or parasites and diseases.

### The apicomplexan parasite–distribution, host specificity and transmission

To date, this apicomplexan pathogen has been identified, using the diagnostic PCR, in four different scallop species from different geographic areas. In addition to the Iceland scallop, king scallop, *Pecten maximus* from UK waters, queen scallop, *A*. *opercularis*, from UK and Faroese waters and sea scallop, *P*. *magellanicus*, off the eastern coast of USA, have been found infected [13,39,40]. Therefore, its geographic distribution is wide and it is non-specific with regards to its pectinid hosts.

Kristmundsson et al. [13] described the morphology of this apicomplexan in three scallop species; the Iceland scallop, the queen scallop and the king scallop, and stated that apparently all life forms necessary for the parasite to complete its life cycle, i.e. merogony, gamogony and sporogony, were present in the scallops. If that is the case the life cycle is monoxenous, i.e. a direct transmission between scallops without an obligate intermediate host being required. However, that can only be fully confirmed by trials where naïve scallops would be exposed to infective sporozoites and samples routinely taken to follow the development of the parasite. Although data on life cycles of apicomplexan infecting marine molluscs are quite scarce, both direct and indirect modes of transmission are known for apicomplexan species, e.g. the heteroxenous *Aggregata* species from cephalopods [42] and monoxenous *Margolisiella* species reported from bivalves and other molluscs [14]. Given that the apicomplexan in the present study is monoxenous, the scallops would most probably get infected via the oral route while unselectively filtering feeding and ingesting infective sporozoites from the environment. This also seems logical considering the observed histology, where massive accumulation of the parasites were observed along the entire gastrointestinal tract. Furthermore, some unidentified parasite stages, which could be developing trophozoites, were seen inside the epithelial cells surrounding the gastrointestinal tract. According to Kristmundsson et al. [13] most of the development seems to take place in the adductor muscle as developing cysts were almost exclusively found there. Two routes of exposure of infective spores seem logical: 1) excretion through the kidney with a subsequent exposure into the environment with feces; 2) from dead decaying scallops which would be especially effective during mass mortality events as well as when the biomass of shells is high. Furthermore, a direct life cycle offers the possibility of an autoinfection which can contribute severely to the proliferation of the pathogen in an infected individual.

Although a direct transmission between scallops seems the most plausible route, the possibility of a two host life cycle cannot be excluded. In that case, the biomass and distribution of proper intermediate hosts would be crucial for an effective dispersal of the pathogen. Furthermore, knowledge on such an intermediate host would be very important to study the epidemiology of the pathogen.

The results show a high prevalence of the infections in all size groups. However, a great difference in the severity of infections is apparent between scallop sizes. Therefore, the scallops seem to get infected at a young age but for some reasons the infections do not seem to intensify in the younger individuals. An age related difference in parasite infections is a well-known phenomenon in case of helminth infections. Larval helminths are thought to remain alive in fish for long periods, often the whole life span of the fish [43]. Due to that, such infections accumulate in the fish over long periods and consequently cause an age related mortality of the fish [43]. However, this is unlikely to be the reason for the difference in the apicomplexan infections in scallops as microparasites, such as apicomplexans, have a much shorter generation time which can be counted in days or weeks but not years [44,45]. Consequently, the explanation for this age difference in the scallops must have some other causes. Age related susceptibility to various infectious agents, such as viruses, are well known in fishes, e.g. the IPNV in which acute infections occur in 1- to 4-mo-old fish and may cause mortalities up to 90% while the susceptibility to the virus decreases with increasing age. Similarly, nodavirus infection predominantly affects the larval or juvenile stages of fish, such as Atlantic halibut (*Hippoglossus hippoglossus*) and Atlantic cod (*Gadus morhua*), in which mortality may be very high [46].

Although purely speculative, this difference could reflect different physiology with age, related to maturity and different immune responses.

### Is the apicomplexan responsible for the collapse of the Icelandic scallop stock?

In addition to the apicomplexan infections, the most common factors considered likely to cause the collapse of the Iceland scallop stock in Breidafjordur in the 2000s were temperature increases, overfishing and food availability [1,21,47]. As in Breidafjordur, unusually high bottom sea temperature was the first factor named as a cause for the mass mortality event in the scallop stock in Hvalfjordur in Iceland in 1983 [3,9]. In 2003 and 2004, the sea temperature in Breidafjordur reached the highest mean in 100 years and the maximum temperature observed in August 2003 (12.2°C) was assumed to exceed the upper temperature tolerance of the species, previously thought to be 10°C [1]. However, subsequent experiments made on scallops from Icelandic waters showed that they can tolerate up to 13°C for at least 3 weeks with no negative effects but considerable mortality was observed at 14°C [20]. Furthermore, the abnormal mortality in the stock had already been observed during the spring survey of the MRI in Iceland in 2001, a period when the sea temperature was considerably lower [21]. This makes temperature increases as the main factor for causing the mortality an unlikely explanation. Similarly, sea temperature alone cannot explain the mass mortality events in the nearby site of Hvalfjordur in 1983 or the fjords and bays in eastern Iceland in the 1990s, as sea temperature there was considerably lower than observed in Breidafjordur during the associated collapses. Based on these findings, Garcia [1] stated that overfishing was left as one of the major factors responsible for the collapse in the scallop stock. However, that is contradictory to the observed mortalities which were unrelated to fishing intensity [3] and the most extensive mortalities during the collapse were actually observed in scallop stocks where none or very minimal fisheries were undertaken [21]. The same argument can be applied to the mass mortality observed in Hvalfjordur in 1983 [9]. When considering lack of food availability as a factor of importance, the abnormal mortality and macroscopic changes were not observed in immature scallops but restricted to the larger ones [3]. For unavailability of food to be considered as a major factor of importance, one would expect the whole populations to be more or less equally affected, regardless of scallop size or maturity.

In general, wild bivalves are poorly studied with respect to apicomplexan parasites and not many examples exist of this group of parasites causing mass mortalities in bivalve populations. The most common apicomplexan species reported from bivalves belong to the genera *Pseudoklossia* and *Margolisiella*. Species of these genera are generally not considered highly pathogenic [14,48–53]. Indeed, this is also the case with *M*. *islandica* which has been described from Iceland scallop in Breidafjordur [14] (unpublished data). The most severe mortalities related to apicomplexan infections in bivalves have occurred among the oyster, *Ostrea chilensis*, on the south coast of New Zealand from late 1985 to 1993 that reduced the population of commercial-sized oysters by 91% [25]. Single or dual infections of two parasite species were identified; a previously unknown apicomplexan species and a known pathogen of oysters. Hine [26] stated that the apicomplexan infection had a significant contribution to this mass mortality event. This apicomplexan, which remains unidentified, also infects two mussel species in New Zealand, *Mytilus galloprovincialois* and *Perna canaliculus*, and is to date considered one of the most serious pathogens of bivalves in New Zealand waters. It primarily infects connective tissues and hampers gonad development [26,54,55]. Therefore, it has been demonstrated that apicomplexan species can cause high mortality rates in wild bivalve populations.

So, is the apicomplexan parasite responsible for the collapse of the Iceland scallop stock in Iceland? Although the prevalence of the apicomplexan infections was high in all sizes of scallops over the study period regardless of the condition of the scallops, the intensity of infections and the prevalence and grades of macroscopic changes of the adductor muscle were in good alignment with the progress of the stock index according to MRI estimation during the 1990s and until 2014 [23]. Furthermore, the extensive histopathological changes associated with the infections indicate that they severely affect the survival of the scallops. The infections mostly affect sexually mature scallops and the pathology observed in the gonads as well as their apparent abnormal development, indicates that the infections seriously impact successful spawning of scallops. This leads to low recruitment, which in turn further contributes to a decrease in the stock. The fact that the recruitment index decreased considerably more than the stock index in these years seems to support this [22].

Since 2009, mostly low level infections have been found in the population, macroscopic changes rarely detected and muscle and gonad condition were normal. Similar findings were reported from UK waters [13] where highly prevalent but low level infections of this apicomplexan were observed in both king and queen scallops but no abnormal condition associated. This might suggest that under normal conditions, low level infections exist in populations of scallops but under hitherto unknown circumstances epidemics can occur. Epidemics, like the one observed in the Iceland scallop, might periodically occur which would make them a major factor in the population dynamics of scallops. What causes such an epidemic is difficult to identify. However, studying the well-known epidemiological triangle, i.e. the host, the infectious agent and the environment is helpful. The infectious agent can be relatively apathogenic, have high pathogenicity, low pathogenicity or even be an opportunistic pathogen. The host can be resistant or have different levels of susceptibility to the pathogen. Then the environment, which can be favourable for the host or unfavourable, in which case it commonly makes the host more susceptible to diseases. With regards to parasites, there can be a good parasite/host homeostasis or this homeostasis can, for some known or unknown reasons, be disrupted. Indeed, knowing these unidentified conditions would be good. However, in an environment like the ocean, there are numerous environmental parameters which could disrupt this homeostasis which in many cases are hard to determine. In fact, one can almost never state that a change in one (or several) particular environmental factor(s) is to blame. In some cases the likeliness is high but statements like these will always be speculative to a certain extent. Even in aquaculture, factors causing subclinical infections (bacteria, viruses, parasites) to develop into a disease outbreak aren´t always known, although these are controlled conditions.

Our findings strongly suggest that the apicomplexan parasite played a major role in the collapse of the Iceland scallop stock in Breidafjordur. Furthermore, compelling evidence exists that indicate that it affects other scallop populations, as similar macroscopic changes and the parasite itself have been identified or observed in association with other mass mortality events in several different scallop species and commercial stocks. However, it seems that these parasite infections can stay at low levels without causing the host any harm. That indicates that some factors, biotic (e.g. host density, presumable intermediate host, different host resistance) or abiotic factors (e.g. temperature, salinity, acidity) cause the parasite to extensively proliferate and cause an epidemic. Which factors could drive such a process are hard to currently ascertain.

Although the recovery of the scallop stock in Breidafjordur has been slow, research has shown clear signs of recovery the last few years.

## Supporting Information

S1 Statistics (DOCX)

S1 Table Sampling times and–sites of all 1493 scallops examined from 2003–2014 (see Fig 1). Abbreviations: AU = autumn; SP = spring; SU = summer.
(DOCX)
