# Supplementary material for: Is an Apicomplexan Parasite Responsible for the Collapse of the Iceland Scallop (Chlamys islandica) Stock?
Source: PLoS One. 2015 Dec 18;10(12):e0144685. doi: 10.1371/journal.pone.0144685 (PMC4684301; doi:10.1371/journal.pone.0144685)
Supplement: S1 Statistics — (DOCX) [file pone.0144685.s001.docx]

**S1-Statistical Analyses**

**Overall**

***Terms and indices***

Ecological terms are according to previous definitions [19]. All statistical tests and plots were performed using RStudio (version 0.98.1062). The Gonad index (GI) and the Muscle Index (MI) are defined in the manuscript.

***On plots and tests***

Notches on boxplots provide an approximate 95% test of the null hypothesis that the true medians are equal: if the notches do not overlap, the medians could be described as statistically significant. However, this is an insufficient way to formally test a hypothesis. Therefore, after determining the normality of the data with the Shapiro-Wilk normality test, multiple groups were compared using ANOVA or the non-parametric Kruskal-Wallis test. When comparing pairs, the non-parametric Wilcoxon test was used.

**Detailed analyses for relevant figures**

**Fig 2A.**


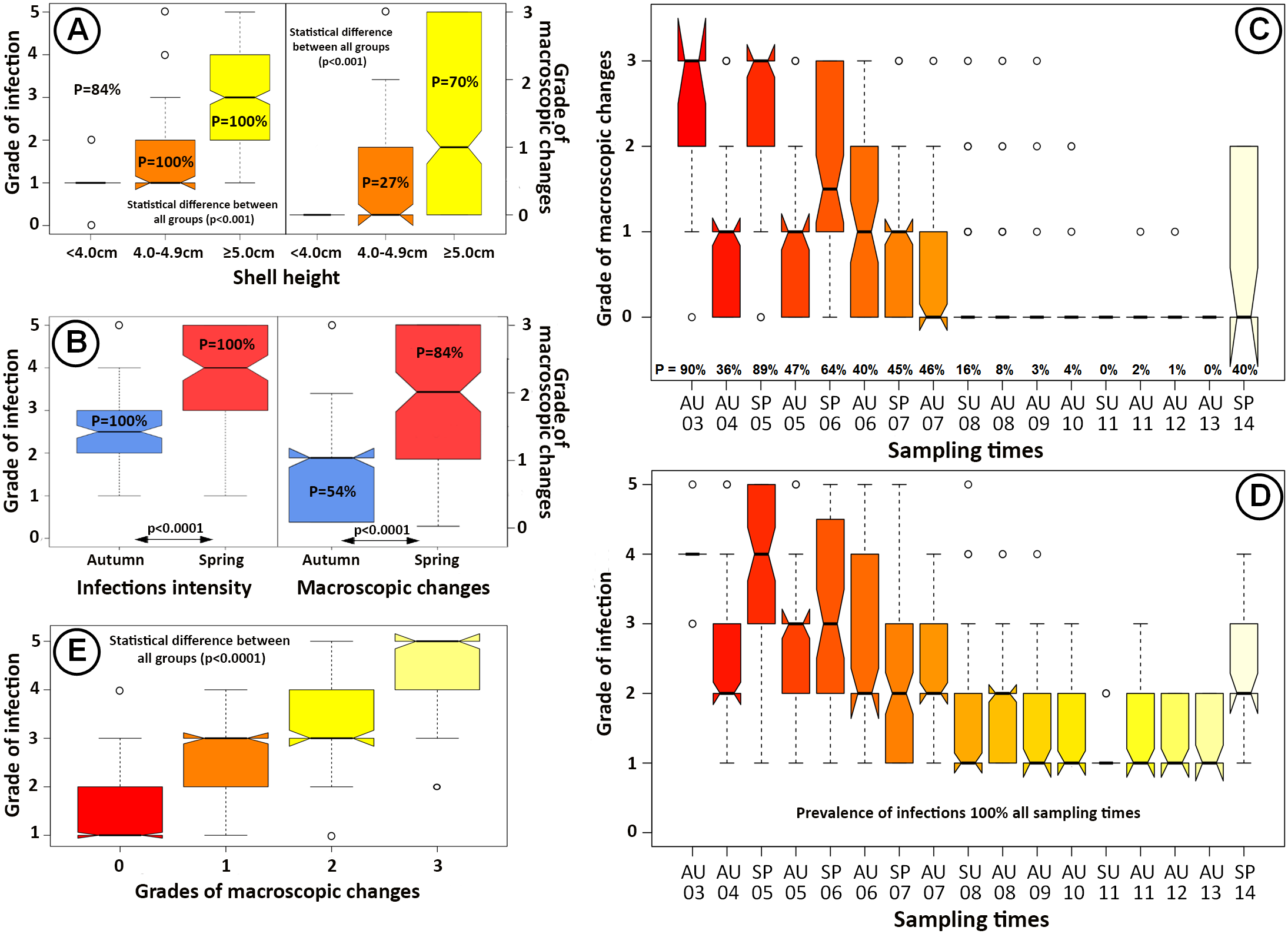


Normality of the data was determined with the Shapiro-Wilk test of normality. In cases of non-normality a non-parametric test was applied.
Grade of infection and microscopic signs were compared between three shell size groups using the non-parametric Kruskal-Wallis test. Results showed a significant difference (p < 0.001) between all size groups in both cases.

**Fig 2B**

| 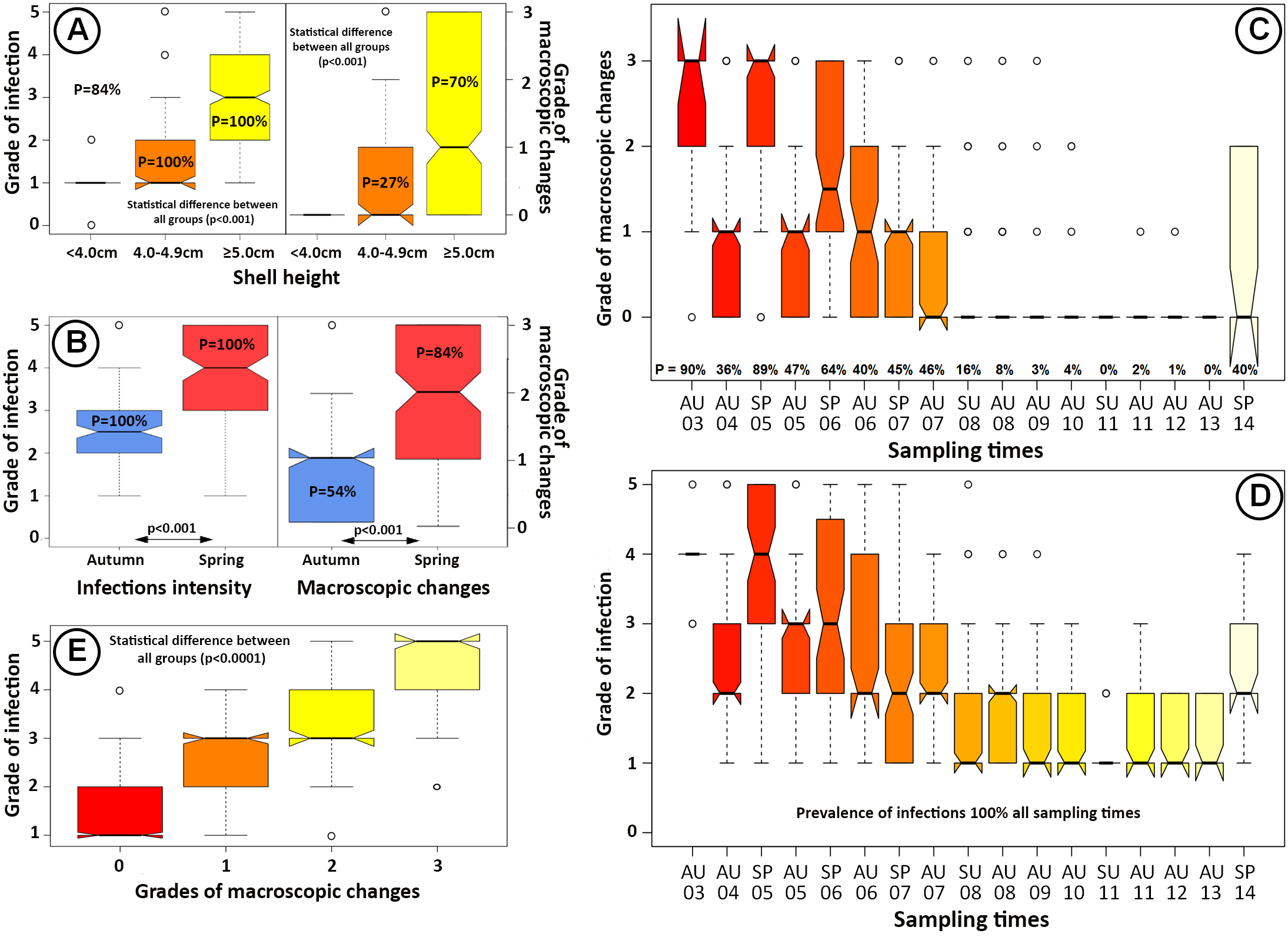 |
| --- |

Seasonal differences of macroscopic changes and the grades of infection were examined separately with the non-parametric Wilcoxon test. The left side of the figure shows a significant difference in grades of infection between spring and autumn during the years 2005 – 2006 (p < 0.001), where the grade of infection is higher in spring than in autumn. The outcome is the same regarding macroscopic changes as significant differences were found between spring and autumn over the years 2005 – 2006 (p < 0.001), where the macroscopic changes were greater during spring.

**Fig 2E**


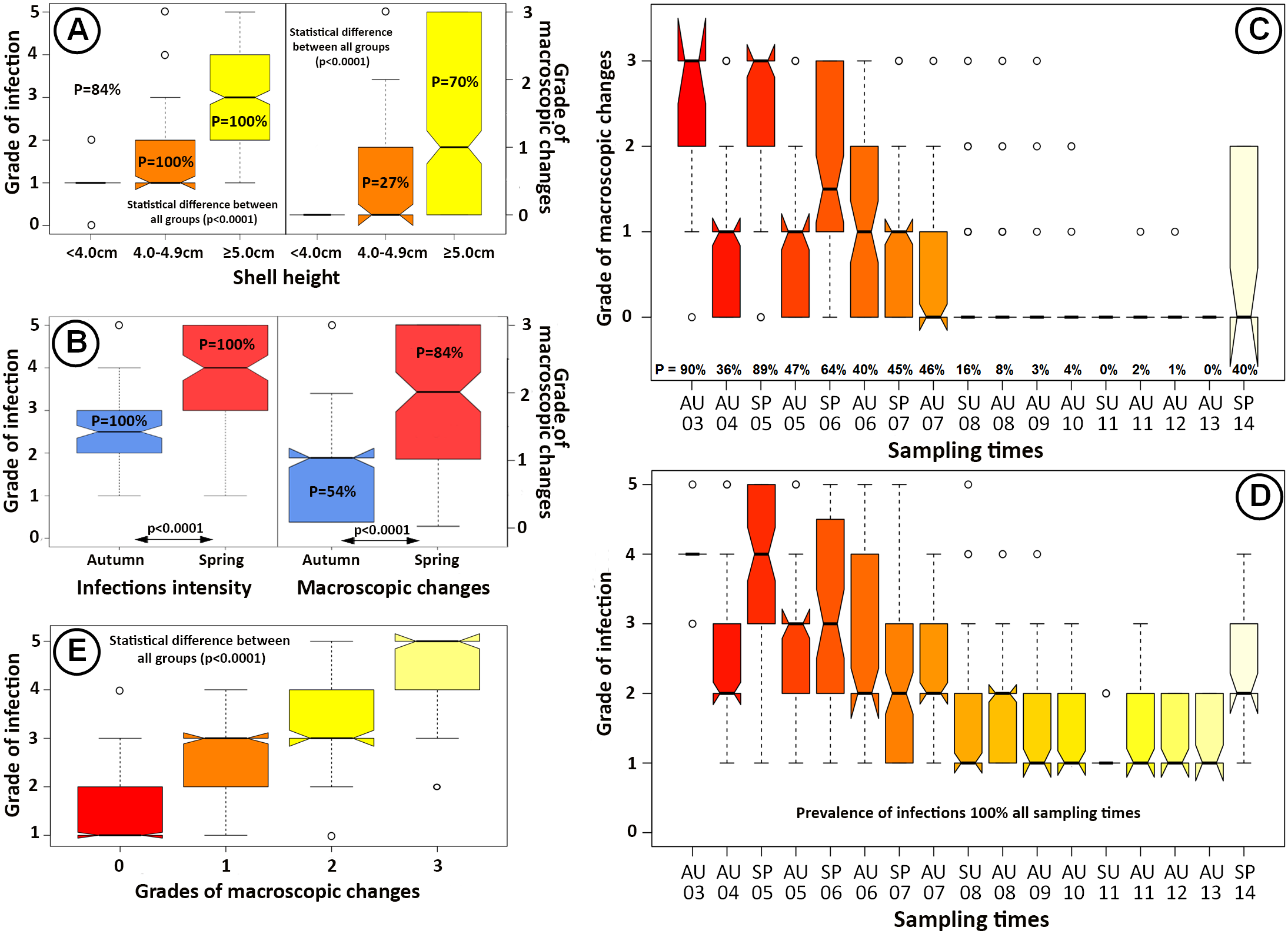


Grades of infection was compared between grades of macroscopic changes with the non-parametric Kruskal-Wallis test. Significant differences were observed between all four groups (p < 0.0001), where the grade of infection was higher with greater macroscopic changes.

**Fig 3B**


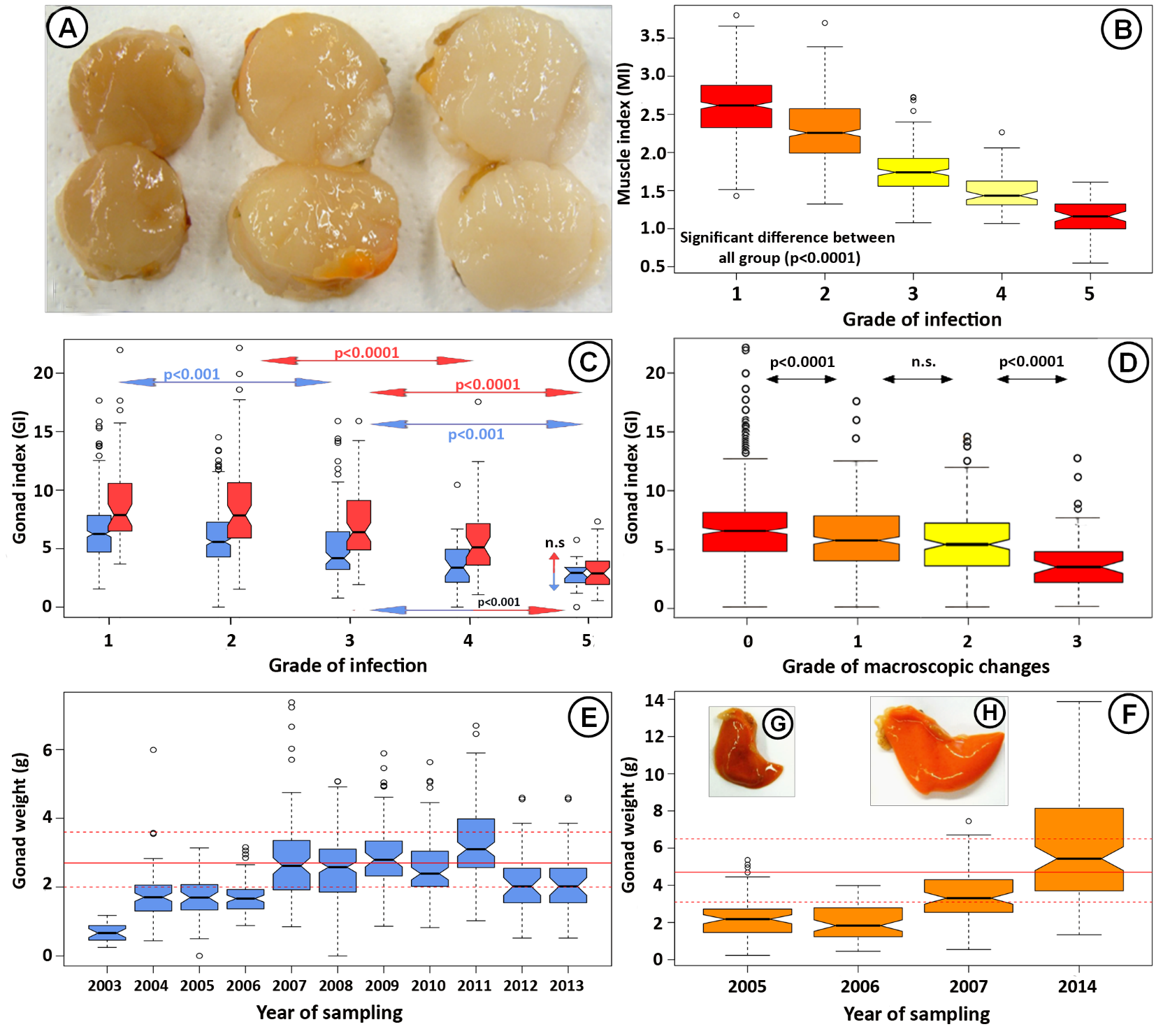


Muscle index was compared between grades of infection, using ANOVA and subsequently, Tukey’s test. Significant differences were found between all five groups (p < 0.0001), where the muscle index was significantly lower in groups with a higher grade of infection.

**Fig 3C**


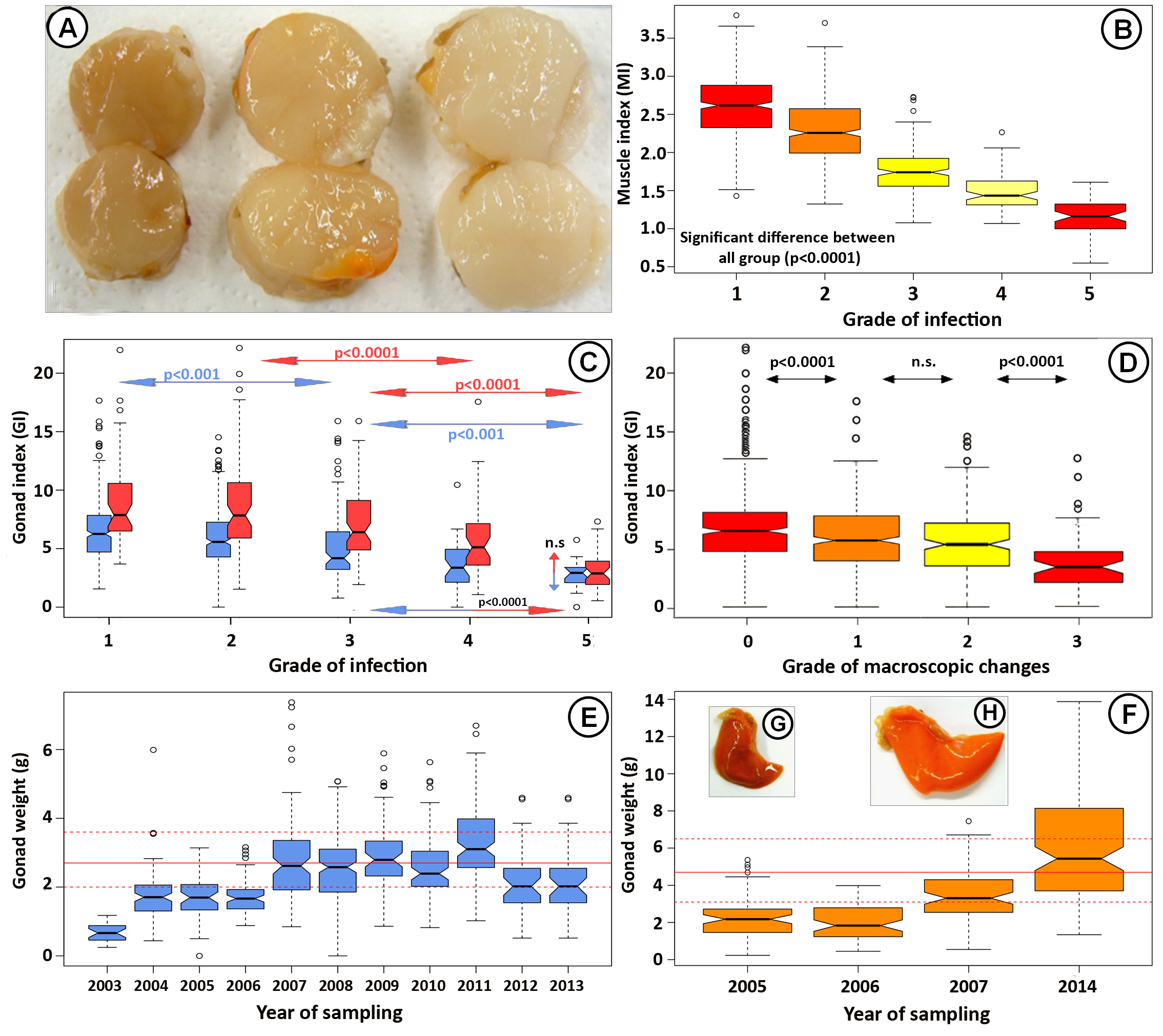


Gonad index was compared between grades of infection for each season, spring (red) and autumn (blue), using the non-parametric Kruskal-Wallis test. Results for autumn showed significant differences between all five groups (p < 0.001), except for groups; 1-2, 3-4 and 4-5 (p > 0.05), which do not significantly differ. Results for spring showed no significant difference between groups; 1-2, 1-3, 2-3 and 3-4 (p > 0.05). Other comparisons were significantly different (p < 0.0001).

When examining each grade of infection between seasons the non-parametric Wilcoxon test was applied. A significant difference was found between seasons for grades 1-4 (p < 0.001). However, the gonad index showed no significant difference between spring and autumn at grade 5 of infection (p > 0.05). In addition, a significant difference was observed between group 5 in spring and groups 1, 2 and 3 (p < 0.0001) in autumn.

**Fig 3D**

| 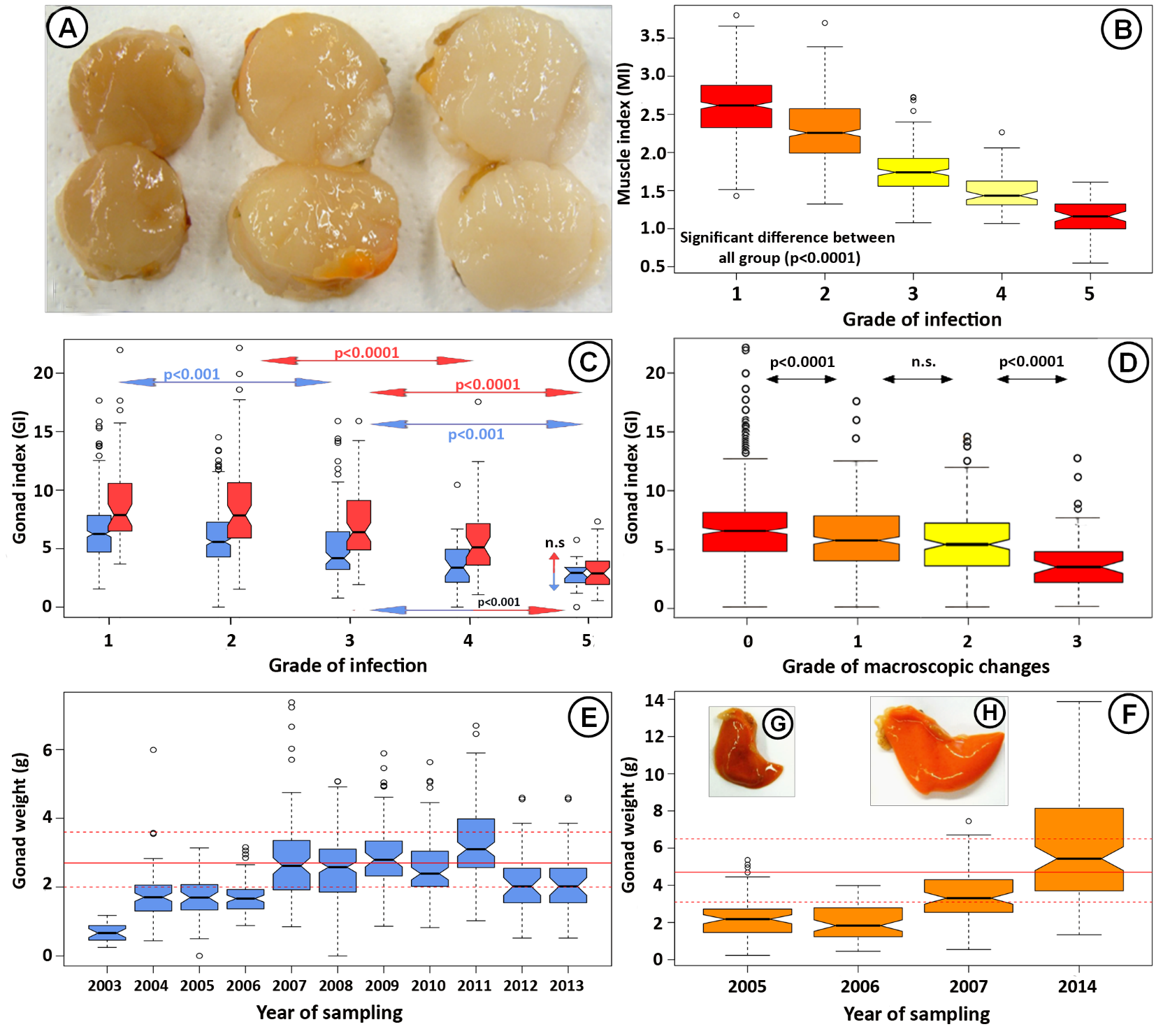 |
| --- |

Gonad index was compared between four groups of macroscopic changes using the non-parametric Kruskal-Wallis test. A significant difference was found between all groups (p < 0.0001), except between groups 1-2 (p > 0.05).
