# Supplementary material for: Is an Apicomplexan Parasite Responsible for the Collapse of the Iceland Scallop (Chlamys islandica) Stock?
Source: PLoS One. 2015 Dec 18;10(12):e0144685. doi: 10.1371/journal.pone.0144685 (PMC4684301; doi:10.1371/journal.pone.0144685)
Supplement: S1 Table — Abbreviations: AU = autumn; SP = spring; SU = summer. (DOCX) [file pone.0144685.s002.docx]

| S1. Sampling times and –sites of all 1493 scallops examined from 2003 – 2014 (see Fig. 1). Abbreviations: AU = autumn; SP = spring; SU = summer. | | | | | | | | | | | | | | | | | | |
| --- | --- | --- | --- | --- | --- | --- | --- | --- | --- | --- | --- | --- | --- | --- | --- | --- | --- | --- |
|  | 2003 | 2004 | 2005 | | 2006 | | 2007 | | 2008 | | 2009 | 2010 | 2011 | | 2012 | 2013 | 2014 |  |
| Sites | AU | AU | SP | AU | SP | AU | SP | AU | SU | AU | AU | AU | SU | AU | AU | AU | SP | Total / site |
| 11 |  | 74 | 50 | 57 | 61 | 60 | 50 | 50 | 60 | 57 | 46 | 31 | 24 | 30 | 30 |  | 30 | 718 |
| 12.1 |  | 83 | 50 | 54 | 31 | 56 | 25 |  |  |  |  |  |  |  |  |  |  | 298 |
| 12.2 |  |  |  |  |  | 26 | 40 | 55 |  | 59 | 14 | 30 |  | 30 | 30 |  |  | 281 |
| 2 |  |  |  |  |  |  |  |  |  | 45 |  |  |  |  |  |  |  | 45 |
| 32.2 | 10 |  | 22 | 5 |  |  |  |  |  |  |  |  |  |  |  | 38 |  | 75 |
| 31 |  |  |  |  |  |  |  |  |  |  |  | 20 |  |  |  |  |  | 20 |
| 33.2 |  |  |  |  |  |  |  |  | 60 |  |  |  |  |  |  |  |  | 60 |
| Total/year | 10 | 157 | 122 | 116 | 92 | 142 | 115 | 105 | 120 | 161 | 60 | 81 | 24 | 60 | 60 | 38 | 30 | 1493 |
| Of those mature | 10 | 96 | 68 | 56 | 64 | 77 | 112 | 105 | 120 | 158 | 60 | 81 | 24 | 60 | 59 | 38 | 30 | 1218 |
